# Neutron Irradiation to Transmute Zinc into Gallium

**DOI:** 10.3390/nano13091487

**Published:** 2023-04-27

**Authors:** M. M. Zeidan, S. Abedrabbo

**Affiliations:** 1 Preparatory Department, Khalifa University, Abu Dhabi P.O. Box 127788, United Arab Emirates; 2 Department of Physics, Khalifa University, Abu Dhabi P.O. Box 127788, United Arab Emirates

**Keywords:** zinc oxide, gallium, neutron, photoluminescence, transmute, zinc isotopes

## Abstract

Modified ^241^Am-Be neutron beams showed an ability to change the optical properties of zinc oxide (ZnO) photoluminescence (PL) spectra by transmuting zinc (Zn) into gallium (Ga) after irradiation. This study investigates the time required by slow neutron irradiation to register the transmutation of the Zn into Ga. Two series of samples from different suppliers hydrothermally (HT) grown by TEW Tokyo Denpa Co. Ltd., Tokyo, Japan, and MTI corporation, China, are irradiated for 6, 12, 18, and 24 days on the Zn-polar face of each sample to specify the relationship between the irradiation intensity and transmutation.

## 1. Introduction

**Figure 1:**
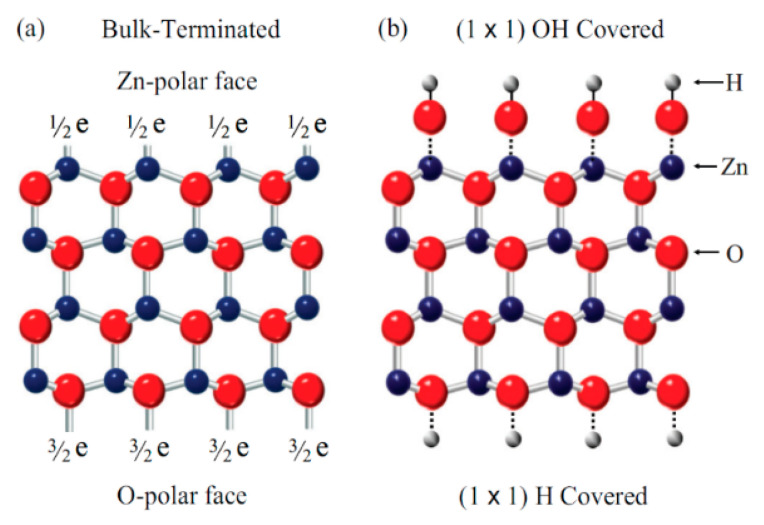
(**a**) Bulk ZnO termination and (**b**) the (**a**) Bulk ZnO termination and (**b**) the (1 × 1) hydroxyl termination of the Zn-polar (0001) and O-polar (0001) faces of ZnO [17].

Zinc oxide (ZnO) is a very attractive material in semiconductors. Presently it is considered a key technological material in semiconductor research. ZnO crystallizes into the wurtzite structure, which is characterized by alternate Zn and O atomic planes (see Figure 1). It has attracted significant attention within the scientific community as a potential semiconductor material. This is due to its properties as a II–VI semiconductor material, including transparency, a wide direct band gap (3.37 eV), large exciton binding energy, ease of growth in the nanostructure form (making ZnO suitable for optoelectronics), transparent electronics, lasing, and sensing for a wide range of applications [1,2,3,4,5,6,7,8,9]. Additionally, ZnO has other properties, including the existence of highly stable excitons, high-electron saturation velocity, the availability of large-area substrates, and relatively low materials costs [7,8,9,10,11,12,13,14,15]. To make high-performance optoelectronic devices on ZnO, it is important to investigate the optical properties of the ZnO. There are different techniques that can be used to study these properties. One of these techniques involves investigating the photoluminescence of ZnO. The photoluminescence of ZnO is intense and shows rich spectra of sharp exciton lines [16,17,18,19,20]. These exciton lines make ZnO an interesting material for optical spectroscopy [17]. On the other hand, investigating the optical properties of irradiated ZnO is important because radiation can significantly alter the optical and electronic properties of materials, including ZnO. In addition, there are many reasons to elaborate on the importance of studying the optical properties of irradiated ZnO, e.g., radiation can cause defects in the crystal structure of ZnO, which can affect its optical and electronic properties. Investigating the optical properties of irradiated ZnO can provide information about the nature and extent of radiation damage, which is important for understanding the behavior of ZnO in radiation environments such as nuclear reactors or space. ZnO is a candidate material for radiation detection due to its radiation-induced luminescence properties. Investigating the optical properties of irradiated ZnO can help researchers design and optimize ZnO-based radiation detectors. ZnO is also used as a material for solar cells, and exposure to radiation can affect its efficiency. Investigating the optical properties of irradiated ZnO can help researchers understand how radiation affects its performance as a solar cell material and how to design more robust and efficient solar cells for use in space or other radiation environments. Investigating the optical properties of irradiated ZnO can also provide insights into the fundamental behavior of materials under radiation, which is important for advancing our understanding of material science and radiation effects.

Semiconductor materials can be modified by irradiation, resulting in changes to their electronic and optical properties. These modifications have a variety of applications, such as the creation of modulated bandgap structures [21,22], alteration of optical properties, and the introduction of optically active impurities. Irradiation also allows for the investigation of fundamental semiconductor properties under extreme conditions in space applications. The group involved in this study has a long history of using irradiation as a technique, having previously performed ion-beam mixing to process modulated bandgap structures and surface modification of Si-substrates to introduce optically active impurities [23,24]. In the present work, we aim to demonstrate the alteration of optical properties in semiconductors through the transmutation of dopant constituents. ZnO is a well-known semiconducting material with high resistivity to radiation compared to other common semiconductor materials, such as Si, GaAs, CdS, and GaN, the latter of which is due to unknown reasons, especially when considering that GaN is a compound with similar lattice properties and a reasonable bond strength [16,25]. There is a shortage in the number of studies regarding the radiation effect on the optical properties of zinc oxide, especially so when considering factors such as room temperature bombardment by high-energy electrons beam, protons, heavy ions or neutrons. Very few researchers have investigated the effect of ionizing radiation on ZnO [18,19]. Excitons are bound states of electrons and holes that can form in a semiconductor when an electron is excited to a higher energy level by absorbing a photon. In ZnO, the excitonic states are strong and well-defined due to the large binding energy of the excitons.

This paper suggests that the irradiation of ZnO crystal bulk with slow neutrons induces changes in the optical properties of the material. Specifically, the near band edge (NBE) photoluminescence (PL) spectra may exhibit an increase in the intensity of the gallium (I_8_) exciton line. This finding is interesting because it suggests that the irradiation of ZnO with slow neutrons alters the band structure of the material, leading to changes in its optical properties. The increased PL intensity of the I_8_ exciton line is due to the creation of defects or impurities in the crystal lattice, which can alter the recombination dynamics of the material.

## 2. Experimental Procedure

### 2.1. Photoluminescence Spectroscopy

A Kimmon Koha Co., Ltd., Tokyo, Japan, helium cadimium (HeCd) laser 325 nm (3.81 eV) line was used to excite the crystals. The photoluminescence (PL) emission was detected using a Spex 1000 M spectrometer purchased from Industries, Inc. 3880 Park Ave., Edison, NJ, USA with a 1200 line/mm holographic grating to disperse the incoming emission light. To complete the detection procedure, a Hamamatsu photomultiplier tube (PMT) procured from Photonics, K. K, Shizuoka Pref. Japan was used. For efficient emission, the sample was mounted inside an Oxford Instruments plc Abingdon, UK liquid He cryostat. The PL intensity signal was registered using a dual channel photon counter connected to LabVIEW software to help plot the PL signal.

### 2.2. Preparing Samples

Two sets of single-crystal ZnO bulk wafers were prepared and cut into multiple pieces (sample) so that each sample was 3 × 3 mm^2^ and 0.5 mm thick. TEW Tokyo Denpa Co. Ltd., (TD), Japan provided the first set. The second set was provided by MTI corporation, China. Both wafers were double-sided, and ZnO was polished and hydrothermally (HT) grown. All of the samples underwent a standardized cleaning process, which involved subjecting them to ultrasonic agitation in organic solvents such as acetone, methanol, and isopropanol. Organic solvents such as acetone, methanol, and isopropanol are also effective for removing oil, grease, and other organic compounds that may be present on the samples. The samples were then thoroughly dried using nitrogen gas (N_2_) to ensure that no residual marks were left behind from the solvents. This final step in the cleaning process helped guarantee that the samples were completely clean and ready for further analysis or processing.

### 2.3. Irradiating Samples

^241^Am-Be with 5.71 MeV and 458 years of half-life was the neutron source. During the decay of the ^241^Am, the alpha particles impinged on the ^9^Be and generated neutrons. ^241^Am-Be sources were in 10 capsules with 1Ci activity, and 1.5 × 10^6^ n/s·cm^2^ neutron flux, placed under a 4.5 cm thick wax block was used to moderate the neutron flux to 7 × 10^4^ n/s·cm^2^. The neutron capsules were distributed under the wax block (10 × 10 cm^2^) and spaced 4.5 cm away from the irradiated samples. Both the spacing and the distribution of the 10 radiating capsules were considered because they allow for the solid angle of the neutron beams to cover the samples with the same flux intensity. The Zn-polar face of the samples was facing down; thus, the Zn isotopes had a bigger chance to interact with the upwards neutrons beam [7].

The samples were grouped according to their duration of exposure to neutron irradiation. Table 1 outlines the specific irradiation schedule for the TD samples, and the MTI crystal bulk samples underwent the same irradiation conditions and exposure time under the identical neutron beams, running in parallel to the TD samples.

## 3. Results

### 3.1. Irradiating the Zn-Polar Face of TD Set

**Figure 2:**
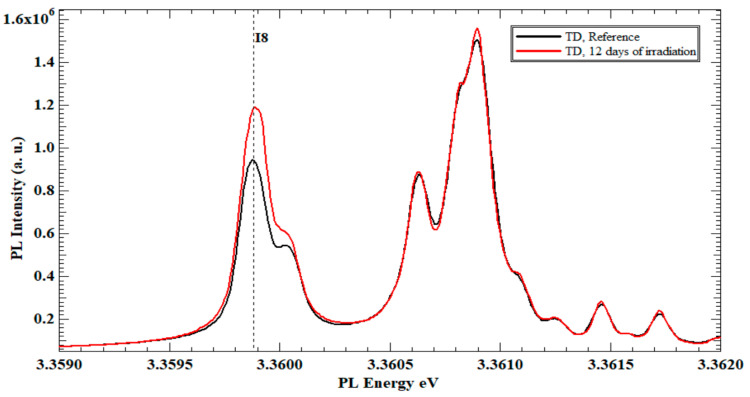
PL spectra at 4K of the Zn-polar face of un-irradiated ZnO single-crystal bulk reference TD sample and TD sample after 12 days of slow neutrons irradiation.

After 6 days of neutron irradiation for sample (A), the PL spectra shows no difference from the PL spectra of the ordinary ZnO single crystal (un-irradiated reference sample). Figure 2 illustrates the Zn polar face PL spectra of sample (B) after 12 days of neutron irradiation compared to the same face of the reference TD ZnO crystal. 

**Figure 3:**
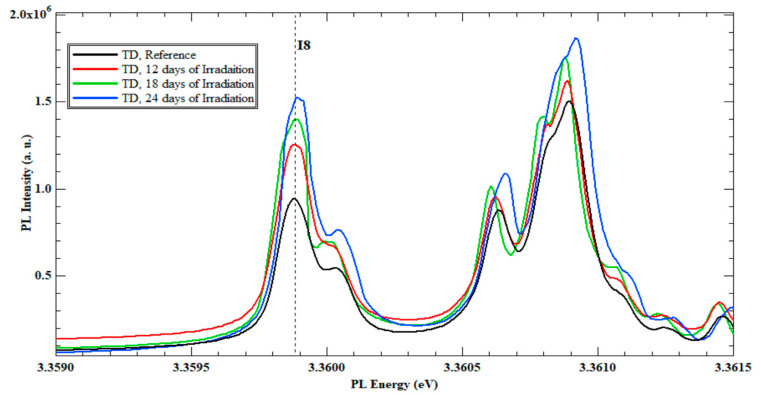
PL spectra’s at 4K of all slow neutrons irradiated Zn-polar face of ZnO single-crystal bulk reference sample and irradiated TD samples after 12, 18 and 24 days.

Exciton line (I8) shows a 26.8% increase in the PL intensity compared to the same exciton line of the reference sample. The exciton line I8 represents the Ga existence in the ZnO crystal structure. Thus, after 12 days of continuous irradiation, the Ga existence increases due to the nuclear reaction between Zn isotopes and neutrons. The PL spectra of samples C (18 days) and D (24 days) present an additional increase in the PL intensity of I8 exciton line. The percentage increase in sample C over sample B is 15.9%, and the increase in sample D over C is 2.5%. Figure 3 shows the TD set PL spectra of the B, C, and D irradiated samples, where the increase of I8 is registered.

### 3.2. Irradiating the Zn-Polar Face of the MTI Set

**Figure 4:**
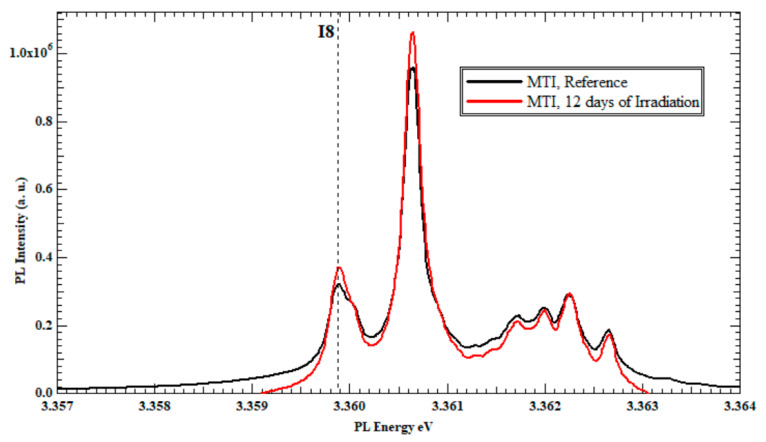
The PL spectra at 4K of the Zn-polar face of un-irradiated ZnO single-crystal bulk MTI and irradiated MTI for 12 days of slow neutrons irradiation.

The results obtained from the irradiation of the MTI set were consistent with and confirmed the results obtained from the TD set. The PL spectrum of sample E exhibited a close resemblance to the PL spectrum of the reference sample, similar to the results obtained for sample A in the TD set (not presented). Figure 4 presents the PL spectra of sample F after 12 days of irradiation. Once again, an increase in the PL intensity of the I_8_ exciton line at the D^o^X region was recorded with a 33% increase compared to the same exciton line from the reference sample.

The other two samples from the MTI set, G and H, reveal additional increases in the PL intensity of the Ga exciton line on each sample, as a 16.2% increase on the PL intensity of the Ga exciton line on sample G over sample F was achieved and Sample H experienced a 6.47% increase in the PL intensity of the I_8_ line over the sample G. 

**Figure 5:**
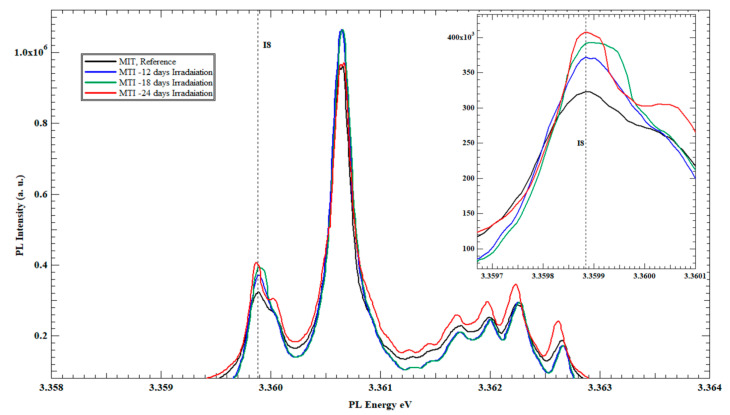
The PL spectra’s at 4K of all slow neutrons irradiated to the Zn-polar face of un-irradiated ZnO single-crystal bulk reference sample and irradiated MTI samples after 12, 18 and 24 days.

Figure 5 illustrates the PL spectra of the samples from the MTI set, F, G and H, in comparison to the reference sample of the same set.

## 4. Discussion

The theory behind irradiating ZnO crystals originated from the fact that we had six Zn isotopes (see Table 2).

When a neutron undergoes radiative capture with a nucleus, the resulting nucleus may become unstable due to the extra neutron it has acquired. Equation (1) describes the nuclear interaction process of radiative capture [27,28,29,30,31,32].

(1) n01 + Xpn+p → Xpn + p + 1 + γ

Some of the newly generated nuclei of this interaction are unstable isotopes that will transmute to another atom depending on their half-lives, according to a beta emission process leading to Ga formation [26,27,28,33,34,35], according to Equation (2).

(2) Xpn + p + 1 →T1/2 Yp + 1n + p + 1 + β−10

Table 3 shows the production of Zn isotopes from the radiative capture.

While irradiating ZnO, the zinc atoms will absorb the neutron and form stable and unstable isotopes (see Table 3). Newly formed ^69^Zn and ^71^Zn gained the most attention in this study because they transmute into gallium (Ga) through β (electron) emission. Indeed, the involved half-lives have a value adequate enough to observe the effects of transmuted atoms.

When Ga is incorporated into the ZnO crystal lattice, it can act as a dopant and introduce additional energy levels within the bandgap of the semiconductor. These energy levels will interact with the excitonic states of ZnO and modify their emission properties. In the photoluminescence (PL) spectroscopy of ZnO grown via the hydrothermal method, Ga is often observed as one of the excitonic lines (I_8_) at 3.3598 eV in the D^o^X region. When the ZnO crystal absorbs a photon and an exciton is formed, the excitonic state can interact with the nearby Ga dopant level and emit a photon at a slightly different energy or wavelength compared to the undoped ZnO. This results in the appearance of an additional excitonic peak in the PL spectrum at the energy level corresponding to the Ga dopant level [32,36,37,38]. Table 3 shows that transmutation into gallium will be achieved when ^68^Zn, ^70^Zn nucleus capture a neutron and form ^69^Zn, ^71^Zn, which then decay to ^69^Ga and ^71^Ga, respectively. 

This interprets the increase in the PL intensity of the I_8_ (Ga) exciton line, as shown in Figure 2 and Figure 4 for the three TD, and MTI-irradiated samples, respectively. In fact, having an initial but unquantified (undeclared from the source) concentration of Ga in the procured ZnO substrates was one instigator to the separate study of TD and MTI samples, while referring to the reference sample from each vendor as the gauging benchmark for I_8_ intensity changes to eliminate the initial Ga intake dependence.

The correlation between the PL intensity increase of the Ga exciton line and neutron irradiation time explains the continuous increase of the gallium concentration in the irradiated ZnO crystals for 12, 18, and 24 days. The percentage increase in the PL intensity supports the argument that the longer the neutrons irradiation period is, the more Zn isotopes transmute into Ga. The fact that the increase rate in I_8_ intensity slows down from 18 to 24 days can be attributed to the quickly depleting initial intake of the reservoir of isotopes ^68^Zn and ^70^Zn (18.8% and 0.6%, respectively), as they become ready to transmute into Ga. It is worth noting that, as more of the Zn-isotopes (^68^Zn and ^70^Zn) in the reservoir transmute into Ga, the new Ga-containing ZnO compound maintains the attractive properties that make it interesting to many applications on top of the enhancement of its optical luminescence properties, as previously stated. As a matter of fact, this study reports for the first time in the literature that ZnO samples from different vendors and of varying initial Ga concentration can have their optical properties, particularly I_8_ excitonic activities, enhanced upon being irradiated while maintaining the attractive properties that stemmed interest in ZnO.

## 5. Conclusions

When slow neutrons are exposed to the Zn-polar face of an un-irradiated ZnO single-crystal bulk, the neutrons get absorbed by the nuclei of zinc atoms in the crystal lattice, causing the nuclei to become unstable and undergo radioactive decay. This process results in the transmutation of some Zn isotopes into Ga isotopes through beta decay. The longer the irradiation time, the more zinc atoms will undergo transmutation, leading to an increasing percentage of gallium atoms in the irradiated ZnO single-crystal bulk. The increase in the PL intensity of the Ga exciton line (I_8_), observed after 12 days of continuous slow neutron irradiation, confirms this transmutation. The increasing intensity of this exciton line in conjunction with longer irradiation time suggests that more Zn isotopes have undergone transmutation, resulting in a higher percentage of Ga in the irradiated crystal bulk. It is worth noting that the process of transmutation requires a sufficient amount of slow neutrons, and a longer irradiation time is needed to achieve a significant increase in the PL intensity of the Ga exciton line.

## Figures and Tables

**Table 1:** The irradiation time series of each sample from the two sets of the un-irradiated ZnO single-crystal bulk.

TD	Irradiation Time (Days)	MTI	Irradiation Time (Days)
A	6	E	6
B	12	F	12
C	18	G	18
D	24	H	24

PL measurement of each sample took place immediately after the irradiation period was complete to examine any optical changes in the PL spectra, especially on the exciton line I8, which represents the Gallium (Ga). An un-irradiated ZnO crystal sample from the original wafer of each set was kept as a PL spectra reference for each set.

**Table 2:** List of Zn isotopes and their abundance [26].

Element	Isotopes	Natural Abundance (%)
Zn	^64^Zn	48.6
	^65^Zn	0.01
	^66^Zn	27.9
	^67^Zn	4.1
	^68^Zn	18.8
	^70^Zn	0.6

**Table 3:** Nuclear reaction of Zn isotopes with neutron and the transmute atom [26].

Isotopes	1st & 2nd Nuclear Reactions	Half-Life (T_1/2_)
^64^Zn	Zn3064 + n1 → Zn3065 + γ Zn3065 + e−1 → Cu2965 + γ	244.26 days
^66^Zn	Zn3066 + n1 → Zn3067 + γ Zn3067 Stable	-
^67^Zn	Zn3067 + n1→Zn3068 + γ Zn3068 Stable	-
^68^Zn	Zn3068 + n1→Zn3069 + γ Zn3069 → Ga3169 + β−1	56.4 min
^70^Zn	Zn3070 + n1 → Zn3071 + γ Zn3071 → Ga3171 + β−1	2.45 min

## Data Availability

All data used in the study are presented in the plots and tables and no further data exist in any database.
